# Roles of meiotic and mitotic cohesin in stem cell gene regulation and genome stability

**DOI:** 10.1016/j.omtn.2026.103100

**Published:** 2026-09-16

**Authors:** Soo Min Cho, Young Eun Kim, Eui-Hwan Choi

**Affiliations:** 1 Department of Biotechnology, Korea National University of Transportation, Chungbuk 27909, Republic of Korea

**Keywords:** MT: clinical applications, embryonic stem cell, cohesin complex, meiotic cohesin, transcription factors, DNA repair, cell cycle, chromosome organization

## Abstract

Cohesin, a ring-shaped SMC complex, mediates sister-chromatid cohesion, DNA repair, and genome folding via loop extrusion. Pluripotent and somatic cells were thought to rely solely on mitotic cohesin (SMC1A, SMC3, RAD21, STAG1/STAG2), while meiosis-specific subunits (SMC1B, REC8, STAG3) were considered germline-restricted. This notion has been challenged, as transcriptomic, proteomic, and chromatin-based evidence shows REC8 and STAG3 are expressed in embryonic stem cells (ESCs), assemble with core subunits like SMC3, and occupy genomic sites distinct from canonical RAD21-cohesin. Here, we compare meiotic and mitotic cohesin in subunit composition, expression, regulatory sensitivities, residence times, and removal mechanisms. We examine how each complex regulates enhancer-promoter communication, couples to the rapid pluripotent cell cycle, and how ESC gene-regulatory landscapes differ from differentiated fibroblasts. We extend the principle that cohesin is recruited by active *cis*-regulatory elements and lineage-determining transcription factors to build cell-type-specific chromatin domains, applying this to meiotic cohesin variants in pluripotent nuclei. Finally, we discuss how REC8/STAG3-containing complexes support replication-fork protection and genome stability during the shortened ESC cell cycle. These findings show that the meiotic-mitotic chromosome organization boundary is permeable, with meiotic cohesin acting as a noncanonical regulator of chromatin architecture and gene expression in pluripotent stem cells.

## Graphical abstract

**Figure:**
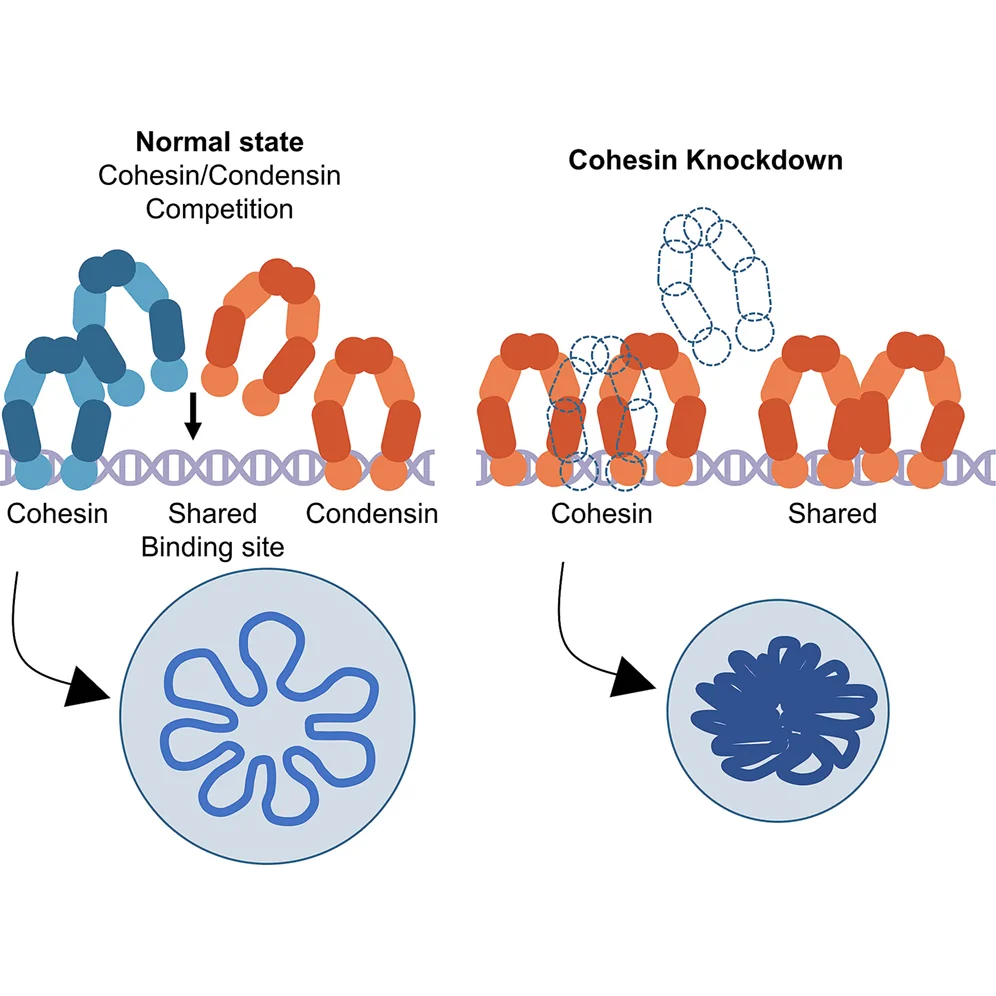

## Teaser

Meiotic-specific cohesins REC8 and STAG3 are increasingly detected in pluripotent stem cells. Choi and colleagues review emerging evidence that these non-canonical complexes shape chromatin architecture, replication dynamics, and genome stability, revealing how meiotic cohesins collaborate with mitotic systems to support pluripotent cell identity and structural demands.

## Introduction

Cohesin complexes are fundamental organizers of eukaryotic chromosomes, mediating sister-chromatid cohesion, facilitating DNA repair, and shaping three-dimensional genome architecture through loop extrusion.^1^^,^^2^^,^^3^ Cohesin is composed of the structural maintenance of chromosome (SMC) heterodimer (SMC3, SMC1A), the kleisin subunit RAD21, and one of several STAG subunits.^4^^,^^5^ This complex supports chromosome segregation during mitosis and contributes to the regulation of gene expression via control of chromatin loops and topologically associating domains (TADs).^6^^,^^7^ In contrast, meiotic cohesin complexes incorporate meiosis-specific subunits such as REC8 and STAG3 and are central to homologous chromosome synapsis, recombination, and the stepwise release of cohesion during meiotic divisions.^8^^,^^9^^,^^10^ The classical view has been that meiotic cohesin expression and function are restricted to germ cells.^8^^,^^11^

Multiple independent lines of evidence report expression of meiotic cohesin components in pluripotent stem cells.^12^^,^^13^ Single-cell transcriptomic surveys detect REC8 and STAG3 transcripts in embryonic stem cell (ESC) populations, and targeted proteomics and immunofluorescence have corroborated protein-level presence and chromatin association in subsets of pluripotent cells.^12^^,^^14^ Importantly, biochemical co-immunoprecipitation (co-IP) experiments indicate that REC8 and STAG3 can assemble into cohesion complex that replace with RAD21-containing structure, implying alternative subunit composition and potentially distinct biochemical behavior.^12^^,^^15^ Chromatin-mapping approaches and chromosome conformation capture methods reveal that binding sites for meiotic cohesin subunits partially overlap with canonical cohesin targets.^16^ However, these factors are also detected at unique genomic loci enriched for developmental regulators and repetitive sequences, suggesting specialized genomic roles.^12^ Functionally, the presence of meiotic cohesin in pluripotent cells has been linked to two broad and non-mutually exclusive activities. First, REC8/STAG3-containing complexes may modulate higher-order chromatin architecture by altering loop extrusion kinetics, boundary formation, or cohesin residence time, thereby influencing promoter-enhancer contacts that sustain pluripotency transcriptional programs.^17^^,^^18^ Second, these complexes appear to contribute to genome-stability pathways during rapid cell proliferation.^19^^,^^20^ Cohesin depletion elevates markers of replication-associated stress and mitotic defects, while recruitment to stalled replication forks intimates a role in fork protection or restart.^13^^,^^19^ A central, and previously underexplored, question is how cohesin, whether built around RAD21 or around the meiotic kleisin REC8, is readout as gene-regulatory information. Cohesin does not bind chromatin uniformly. Rather, it is recruited and repositioned by active *cis*-regulatory elements and lineage-determining transcription factors, and its loop-extrusion activity is tuned by CTCF, WAPL, PDS5, and Sororin to generate cell-type-specific enhancer-promoter interaction.^21^^,^^22^^,^^23^ Whether meiotic cohesin variants are subject to the same logic, and how their incorporation alters the gene-regulatory output of pluripotent chromatin, is the primary focus of this review.^24^

The mechanistic basis for these activities likely involves differential interactions with cohesin regulators, cell-cycle-dependent expression dynamics, and post-translational modifications that reprogram meiotic proteins for somatic and pluripotent functions.^15^^,^^25^ In this review, we compare meiotic and mitotic cohesin and assess the molecular, genomic, and cellular evidence for meiotic cohesin function in pluripotent stem cells.^26^ We first summarize cohesin structure and regulation and then describe the meiotic subunits. We next provide a dedicated comparative analysis of mitotic versus meiotic cohesin, evaluate their expression and roles in ESCs, and then focus on gene regulation by both complexes and on the cooperation of cohesin with transcription factors at cell-type-specific enhancers. Finally, we discuss replication-coupled genome-stability functions and outstanding mechanistic questions.

## Structure and regulation of the cohesin complex

### Structure of the cohesin complex

The cohesin complex is a highly conserved multiprotein assembly that plays a central role in chromosome segregation, genome stability, and three-dimensional genome organization.^1^^,^^2^ Cohesin belongs to the SMCs protein family and functions as a ring-shaped molecular complex capable of topologically entrapping DNA.^2^^,^^27^ This structural arrangement enables cohesin to physically tether sister chromatids and regulate higher-order chromatin architecture (Figure 1A).^3^

**Figure 1:**
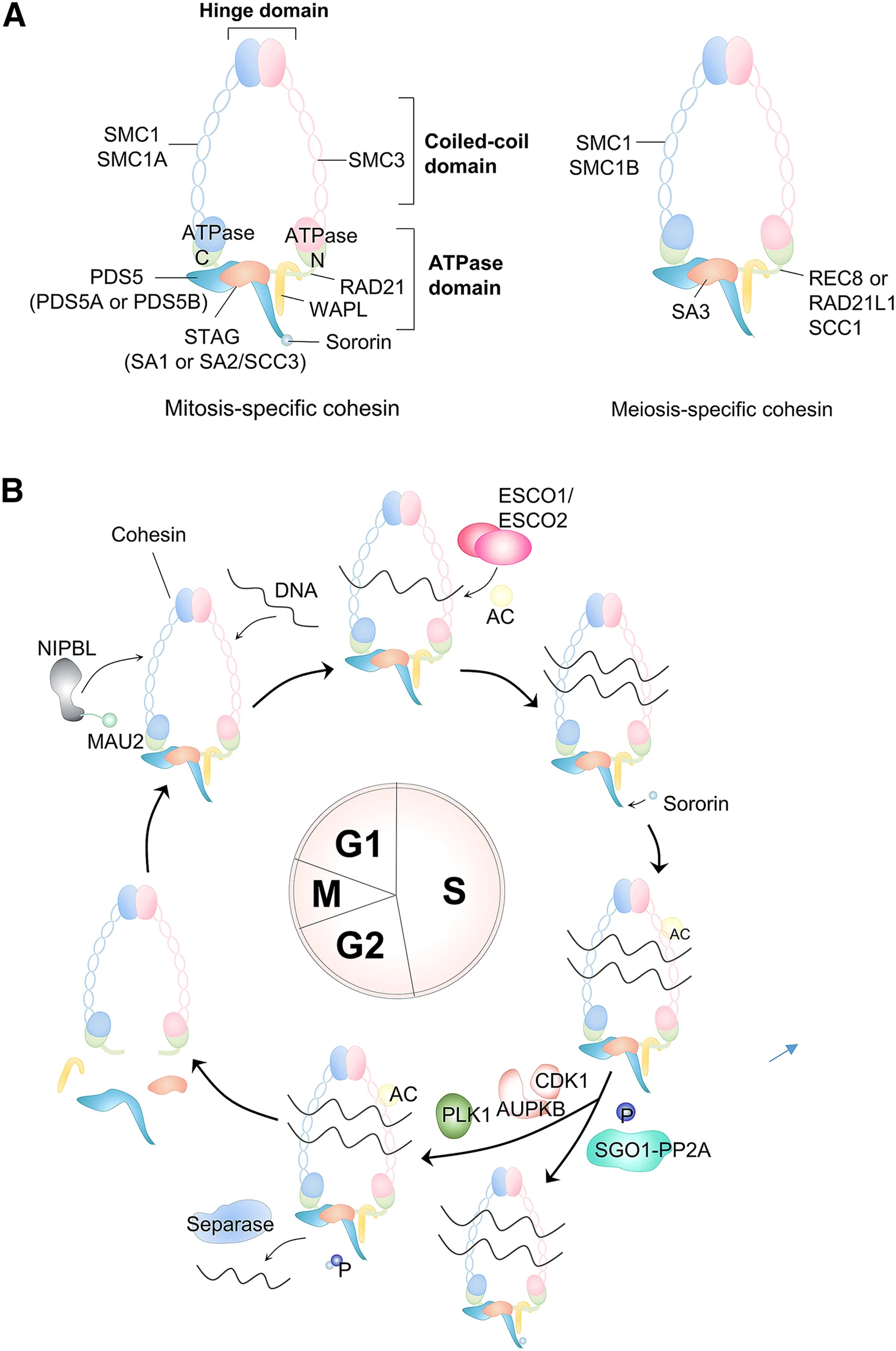
Mitotic and meiotic cohesin complex structures (A) Schematic representation of the mitosis-specific cohesin complex. The canonical cohesin ring consists of SMC1A and SMC3 heterodimer connected by the kleisin subunit RAD21 and associated with STAG1/2. Regulatory factors including PDS5, WAPL, and Sororin dynamically modulate cohesin stability and chromatin association. (B) Schematic representation of the meiosis-specific cohesin complex. In this complex, meiosis-specific subunits such as SMC1B, REC8, and STAG3 replace their mitotic counterparts. These alternative components generate cohesin complexes with distinct regulatory properties and functions in chromosome architecture and genome stability.

The core cohesin complex consists of four primary subunits. SMC3, SMC1A, RAD21, STAG1, and STAG2. These proteins form a tripartite ring-like structure capable of embracing chromatin fibers.^4^^,^^28^ SMC1A and SMC3 are long coiled-coil proteins that form an antiparallel heterodimer through their hinge domains located at the center of the molecule. Each SMC protein contains an ATPase head domain at its N- and C-termini, which are brought together through folding of the coiled-coil region.^28^ These ATPase head domains belong to the ATP-binding cassette (ABC) family and are critical for cohesin loading, DNA entrapment, and structural remodeling.^28^^,^^29^ The kleisin subunit RAD21 bridges the ATPase head domains of SMC1A and SMC3, thereby closing the cohesin ring.^4^^,^^30^ RAD21 binds to SMC1A at its C-terminal region and to SMC3 at its N-terminal region, effectively linking the two SMC proteins into a stable tripartite structure. This arrangement forms a large proteinaceous ring that can encircle DNA molecules (Figure 1A).^30^

The fourth core component of the complex is the HEAT-repeat protein STAG1 or STAG2 (known as SA1 or SA2).^5^^,^^31^ These proteins interact with RAD21 and contribute to stabilizing cohesin binding to chromatin.^30^ STAG proteins are also thought to facilitate interactions with regulatory factors and chromatin-associated proteins, thereby influencing cohesin’s genomic localization and functional specificity.^31^ Although both STAG1 and STAG2-containing complexes share similar structural architectures, they exhibit partially distinct chromatin binding patterns and regulatory roles.^5^^,^^16^ STAG1-containing cohesin complexes are frequently associated with chromatin boundaries and long-range chromatin loops, whereas STAG2-containing complexes are more commonly involved in dynamic regulatory interactions at gene promoters and enhancers (Figure 1A).^5^^,^^31^ These differences suggest that alternative STAG subunits may confer functional specialization to cohesin complexes.^32^

### Cohesin loading and establishment of sister chromatid cohesion

The loading of cohesin onto chromatin occurs primarily during late telophase and early G1 phase of the cell cycle.^25^ This process is mediated by the cohesin loader complex composed of NIPBL (Nipped-B-like protein) and its partner MAU2.^33^ The NIPBL-MAU2 complex facilitates the opening of the cohesin ring and promotes its topological loading onto DNA in an ATP-dependent manner.^28^^,^^34^ Once loaded onto chromatin, cohesin can perform multiple functions depending on the cell cycle stage. During G1 phase, cohesin contributes to the establishment of chromatin loops and higher-order genome organization.^35^ However, the physical linkage between sister chromatids is not yet established at this stage because DNA replication has not occurred. Sister chromatid cohesion is established during S-phase, when DNA replication generates duplicated chromatids.^25^ During replication, cohesin complexes capture the newly formed sister chromatids within the cohesin ring.^19^ This process is facilitated by the acetylation of SMC3, which is catalyzed by the acetyltransferases ESCO1 and ESCO2 (Figure 1B).^29^ Acetylation of SMC3 stabilizes cohesin on chromatin and prevents premature dissociation mediated by negative regulators such as WAPL.^29^^,^^36^ The establishment of stable cohesion is further reinforced by the recruitment of Sororin, a regulatory protein that antagonizes WAPL activity.^25^ Sororin binds to the cohesin complex and promotes stable sister chromatid cohesion by inhibiting cohesin release factors (Figure 1B).^15^

### Maintenance and dynamic regulation of cohesin during interphase

Following cohesion establishment during S-phase, cohesin complexes maintain sister chromatid cohesion throughout G2 phase until the onset of mitosis (Figure 1B).^25^ During this period, cohesin complexes continue to regulate chromatin architecture and gene expression.^23^ Cohesin function during interphase is highly dynamic and tightly regulated by several sub-proteins.^21^^,^^23^ The proteins PDS5A and PDS5B interact with cohesin and play dual roles in regulating both cohesion stability and cohesin turnover.^23^ PDS5 proteins can recruit either Sororin or WAPL, thereby influencing whether cohesin remains bound to chromatin or is released.^15^^,^^23^ Another important regulator is WAPL, which promotes the opening of the cohesin ring and facilitates cohesin release from chromatin.^21^^,^^36^ WAPL-mediated release is an essential mechanism that allows cohesin complexes to dynamically reposition along chromatin and maintain appropriate genome architecture.^21^

This dynamic loading and unloading process is particularly important for cohesin’s role in loop extrusion, a mechanism by which cohesin progressively enlarges chromatin loops to organize the genome into TADs.^35^^,^^37^ Loop extrusion is believed to be driven by the ATPase activity of the SMC1A-SMC3 head domains.^38^^,^^39^

### Cohesin removal during mitosis

The removal of cohesin from chromosomes is a tightly regulated process that ensures accurate chromosome segregation during mitosis.^40^ Cohesin removal occurs in two major steps during mitotic progression. The first step takes place during prophase, when a large fraction of cohesin is removed from chromosome arms through prophase pathway.^41^ This pathway is mediated by WAPL and other regulatory factors and occurs independently of proteolytic cleavage.^41^ During this stage, cohesin complexes dissociate from chromosome arms while remaining protected at centromeres. Centromeric cohesin is protected by the Shugoshin (SGO1)-PP2A complex, which prevents premature removal of cohesin at the centromere.^42^ This protection is essential for maintaining sister chromatid cohesion until the appropriate stage of mitosis (Figure 1B).^42^^,^^43^ The final removal of cohesin occurs at the transition from metaphase to anaphase. At this stage, the protease separase becomes activated following the degradation of securin and cyclin B.^44^ Activated separase cleaves the RAD21 subunit of cohesin, thereby opening the cohesin ring and releasing the physical linkage between sister chromatids.^45^^,^^46^ The cleavage of RAD21 triggers the rapid separation of sister chromatids, allowing them to be pulled toward opposite spindle poles.^4^ This step is essential for accurate chromosome segregation and completion of cell division.^40^

### Functional modification of cohesin complexes

In addition to its established role in sister chromatid cohesion, cohesin participates in numerous cellular processes including transcriptional regulation, DNA repair, and maintenance of genome stability.^22^^,^^47^ Cohesin’s role in chromatin architecture has become particularly prominent with the discovery that cohesin works together with chromatin boundary factors such as CTCF to organize the genome into loop domains.^7^^,^^22^^,^^23^ The ability of cohesin to dynamically load, translocate, and unload along chromatin allows it to function as a central regulator of genome topology.^17^^,^^21^ Dysregulation of cohesin components or their regulatory factors can therefore lead to profound defects in chromosome segregation and gene regulation.^48^ Indeed, mutations in cohesin subunits or cohesin regulatory proteins have been linked to a variety of developmental disorders and cancers.^47^^,^^49^^,^^50^^,^^51^ These observations reveal the importance of cohesin as a key architectural regulator of the genome.^52^

## Meiotic cohesin components

In addition to the canonical mitotic cohesin complex, specialized cohesin variants function during meiosis to ensure proper homologous chromosome pairing and segregation.^8^ These meiotic cohesin complexes include meiosis-specific subunits such as SMC1B, REC8, and STAG3, which replace their mitotic counterparts.^8^^,^^9^

REC8 acts as a meiosis-specific kleisin subunit that replaces RAD21 and plays a critical role in maintaining cohesion between sister chromatids during the prolonged stages of meiotic prophase.^9^^,^^53^ Unlike RAD21, which is removed during mitosis in a relatively synchronized manner, REC8 is differentially protected along chromosome arms and centromeres to allow the stepwise separation of homologous chromosomes during meiosis I and sister chromatids during meiosis II.^11^^,^^42^ STAG3 is another key component of meiotic cohesin complexes and contributes to the stabilization and proper localization of REC8-containing cohesin complexes along chromosome axes.^10^ Genetic studies have demonstrated that loss of STAG3 leads to severe defects in synapsis, recombination, and chromosome segregation, highlighting its essential role in meiotic chromosome dynamics.^10^^,^^54^ SMC1B, a paralog of SMC1A, also functions specifically during meiosis and is required for the establishment and maintenance of meiotic chromosome architecture.^55^^,^^56^ Together, SMC1B, REC8, and STAG3 form a specialized cohesin complex that ensures accurate chromosome pairing and recombination during gametogenesis.^8^^,^^57^ Although these proteins were originally considered strictly meiosis-specific, emerging evidence suggests that their expression may occur in certain non-meiotic contexts, raising questions about potential roles outside the germline.^12^^,^^15^^,^^58^

## Comparative analysis of mitotic and meiotic cohesin explaining shared architecture and divergent biology

Both are ring-structured SMC assemblies that entrap DNA and extrude loops, yet they differ in subunit identity, expression timing, regulatory wiring, residence time, and biological output (Table 1).^3^^,^^59^ Recent structural and single-molecule work has clarified that the loop-extrusion machinery is broadly conserved across SMC complexes, making the subunit-level substitutions in meiotic cohesin all the more informative for understanding functional differentiation.^38^^,^^39^^,^^60^ Beyond their compositional differences, the two complexes also occupy distinct functional niches along the chromosome. Specifically, mitotic cohesin establishes sister-chromatid cohesion and regulate interphase TADs, whereas meiotic cohesin additionally builds the proteinaceous chromosome axis that scaffolds the synaptonemal complex and channels recombination intermediates toward crossover formation.^9^^,^^61^ This dual mechanical and architectural role means that meiotic cohesin variants are not simply cohesion factors operating in a different cellular process, but components of a structurally chromatin remodeling machinery.^53^ Understanding how these two cohesin identities are segregated, and where they overlap, as in pluripotent cells, therefore has direct bearing on how ectopic or aberrant meiotic-cohesin expression might be expected to agitate genome architecture and stability in non-germline.^58^ This question is of particular relevance to regenerative medicine and oncology, where loss of cohesin fidelity is increasingly recognized as a driver of genomic instability.^47^^,^^49^

**Table 1:** Comparative features of mitotic and meiotic cohesin complexes

Feature	Mitotic cohesin	Meiotic cohesin
SMC heterodimer	SMC1A-SMC3	SMC1B-SMC3-SMC1A variants
Kleisin subunit	RAD21	REC8, RAD21L
HEAT/STAG subunit	STAG1/STAG2	STAG3
Primary expression context	all proliferating somatic and pluripotent cells; constitutive	germline meiosis; partially active in ESCs, repressed in MEFs
Cell-cycle/stage timing	load G1; cohesion in S; maintained G2	induced at meiotic entry; persists through pachytene
Loading factor	NIPBL-MAU2	NIPBL-MAU2
Removal mechanism	prophase pathway (arms) + separase cleavage of RAD21 (anaphase)	two-step: SGO2-PP2A protection; cleavage of REC8 on arms (anaphase I) then centromeres (anaphase II)
Residence/loop length	dynamic; shorter regulatory loops	longer residence; extended axis-loop architecture
Regulator sensitivity	STAG1 vs. STAG2 differ in NIPBL/WAPL dependence	STAG3-dependent; altered WAPL/Sororin release kinetics
Canonical biological output	sister-chromatid cohesion, TAD/loop formation, enhancer–promoter regulation	axis-loop formation, synapsis, recombination
Output when present in ESCs	pluripotency-associated genome folding, gene regulation	replication-fork protection, genome stability, fine-tuning of pluripotency programs

### Subunit composition and structural homology

Mitotic cohesin is composed of the SMC1A-SMC3 heterodimer, the kleisin RAD21, and STAG1 or STAG2.^4^^,^^5^ Meiotic cohesin substitutes the kleisin REC8, the SMC1A paralog SMC1B, and the STAG paralog STAG3.^8^^,^^56^ These replacements preserve the three-part ring shape, REC8, like RAD21, bridges the SMC ATPase heads, and STAG3, like STAG1/2, is a HEAT-repeat subunit that contacts the kleisin, but they alter surface chemistry and regulator-binding interfaces.^10^^,^^53^ Cryo-electron microscopy (EM) and crystallographic studies of cohesin subcomplexes indicate that the kleisin N- and C-terminal domains dock onto the SMC3 and SMC1 ATPase heads, respectively, through interfaces that are structurally conserved between RAD21 and REC8 despite limited primary-sequence identity.^28^ This preservation reflects a strong selective pressure to maintain the specific structural requirements for ATP-dependent ring opening and DNA entrapment.^62^ However, REC8 carries meiosis-specific sequence insertions, including motifs implicated in recruitment of the SGO1-PP2A axis, that are absent from RAD21 and that discuss the differential centromeric protection.^42^^,^^53^ Likewise, STAG3 diverges from STAG1/2 most markedly in loop regions thought to mediate contacts with NIPBL-MAU2 and with WAPL, suggesting that subunit substitution is not merely a matter of paralog redundancy but a mechanism for rewiring the entire regulatory circuit that governs loading, stabilization and release.^15^^,^^34^

Importantly, the various meiotic subunits can associate combinatorially, generating a far larger diversity of cohesin species than the mitotic complex affords.^8^ This combinatorial diversity is thought to underlie the layered, sequential roles of meiotic cohesin during prophase I. RAD21L-containing complexes are implicated in early axis formation and homolog pairing, while REC8-containing complexes are more closely tied to sister-chromatid cohesion and to the protection of centromeric cohesion through meiosis I.^9^^,^^11^ Whether analogous combinatorial logic operates in non-germline cells expressing meiotic subunits remains unexplored. In ESCs, REC8, and STAG3 are recovered in SMC3-containing complexes, and STAG3 is required to stabilize nuclear REC8, indicating that competent hybrid rings, meiotic kleisin/STAG paired with otherwise canonical SMC and regulatory machinery, can form outside meiosis.^12^^,^^15^ This raises a structurally important question. Because SMC1B is largely germline-restricted, hybrid complexes in ESCs likely pair REC8 and STAG3 with the canonical SMC1A-SMC3 heterodimer rather than the meiosis-specific SMC1B-containing core.^55^^,^^56^ This implies a chimeric architecture whose biophysical properties, such as residence time, extrusion processivity, and mechanical stiffness, have not yet been directly measured, representing a clear gap for future single-molecule and structural studies.^18^^,^^63^

### Cell-cycle- and stage-specific expression dynamics

Mitotic cohesin subunits are expressed constitutively across the proliferating-cell cycle, peaking with loading in late telophase/G1 and cohesion establishment in S-phase.^25^ Their transcription is governed largely by general cell-cycle transcription factor networks rather than by lineage- or stage-specific enhancers, consistent with a housekeeping role in genome maintenance.^4^ Meiotic subunits, by contrast, show a restricted, developmentally staged expression REC8, STAG3, and SMC1B are strongly induced at meiotic entry (leptotene, zygotene), persist through pachytene to organize the chromosome axis, and are stepwise removed across meiosis I and II.^8^^,^^11^ This induction coincides with, and is at least partly driven by, meiosis-specific transcriptional processes, positioning meiotic-cohesin genes within a broader regulatory module dedicated to meiotic differentiation.^64^^,^^65^

The unexpected observation is that this meiotic program is partially active in pluripotent cells. ESCs express REC8 and STAG3 transcripts and protein, whereas differentiated mouse embryonic fibroblasts (MEFs) largely suppress them (Figure 2).^12^^,^^13^ This is consistent with the well-described phenomenon of partial activation of meiotic and meiotic gene modules in pluripotent stem cells, which share with germ cells an open, hypomethylated chromatin state and reactivation of select meiotic transcription factors.^66^^,^^67^ Single cell analyses suggest that meiotic-cohesin expression in ESCs is heterogeneous and state-dependent, consistent with a permissive but transcriptionally tuned program that is silenced upon differentiation.^14^ This heterogeneity raises the possibility that meiotic-cohesin expression marks, or even contributes to, substates of pluripotency analogous to the 2-cell-like or totipotent-like states already linked to transient meiotic- and repeat-element reactivation, a hypothesis that single cell multi-omic profiling could directly test.^66^ Thus, the two complexes differ not only in their composition but also in their timing and cellular states of expression.^25^ Mitotic cohesin expression is essentially binary, whereas meiotic-cohesin expression behaves as a graded, cell-fate-specific variable whose functional threshold for engaging chromatin in ESCs is not yet defined.^24^

**Figure 2:**
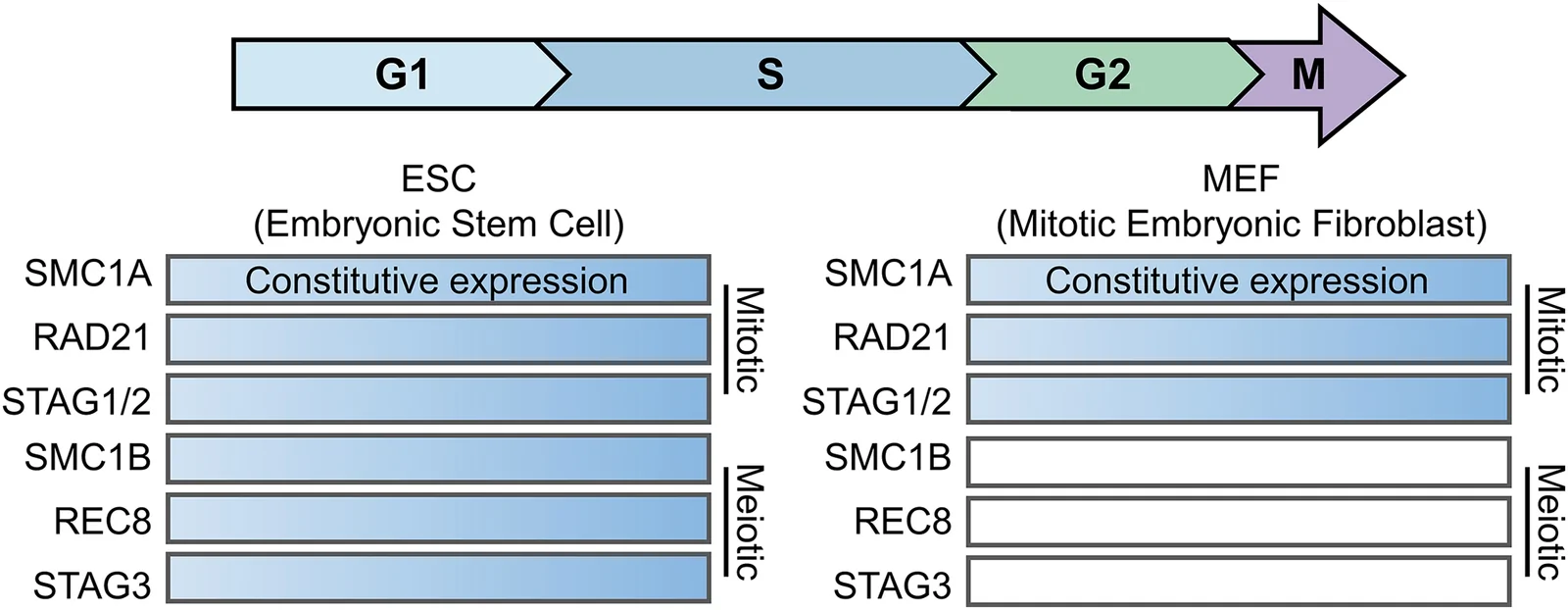
Expression patterns of mitotic and meiotic cohesin subunits in ESCs and MEFs Schematic showing the cell cycle stages (G1, S, G2, and M) and expression of mitotic (SMC1A, RAD21, and STAG1/2) and meiotic (SMC1B, REC8, and STAG3) cohesin subunits in ESCs and MEFs. ESCs constitutively express both mitotic and meiotic cohesin subunits throughout the cell cycle, whereas MEFs express only mitotic cohesin subunits.

### Divergent regulatory sensitivities

Although meiotic and mitotic cohesin share the key regulators NIPBL-MAU2, PDS5A/B, WAPL, Sororin and the ESCO acetyltransferases, their sensitivities differ.^23^^,^^29^ Mitotic cohesin-STAG1 and cohesin-STAG2 already display distinct NIPBL requirements and chromatin behaviors, establishing that STAG identity tunes regulator dependence; by extension, STAG3 is expected to impose its own regulatory logic.^5^^,^^34^ Studies in somatic systems show that REC8-containing cohesin requires STAG3 to function and remains subject to WAPL and Sororin, but with altered release kinetics relative to RAD21 cohesin.^15^ This altered kinetics is consistent with reports that meiotic cohesin complexes display longer chromatin residence times than their mitotic counterparts *in vivo*.^18^^,^^68^ This property is thought to be important for maintaining persistent cohesion across the extended prophase I arrest that characterizes meiosis, but its molecular basis, whether reduced WAPL affinity, enhanced Sororin-mediated antagonism of WAPL, or intrinsic differences in ring dynamics, remains incompletely resolved.^15^^,^^36^ Importantly, the removal mechanism diverges between the two processes. Mitotic cohesin is largely stripped from arms by the prophase pathway and then cleaved at RAD21 by separase in a single anaphase step, whereas REC8 is protected by SGO2-PP2A at centromeres and cleaved in two steps, on arms at anaphase I and at centromeres at anaphase II, to enforce the reductional-then-equational segregation pattern (Figure 3).^41^^,^^42^^,^^69^ This two-step release depends on a spatially restricted pool of PP2A-B56 that counteracts Aurora B/C-mediated phosphorylation of SGO2 substrates at centromeres while allowing phosphorylation-driven cohesin loss to proceed unimpeded along chromosome arms, a spatial regulatory logic that has no clear counterpart in the mitotic prophase pathway.^42^ Separase itself shows differential cleavage efficiency toward REC8 versus RAD21 *in vitro*, which may further contribute to the temporal separation between arm and centromeric cohesin loss during meiosis I and II (Figures 4A and 4B).^45^^,^^46^

**Figure 3:**
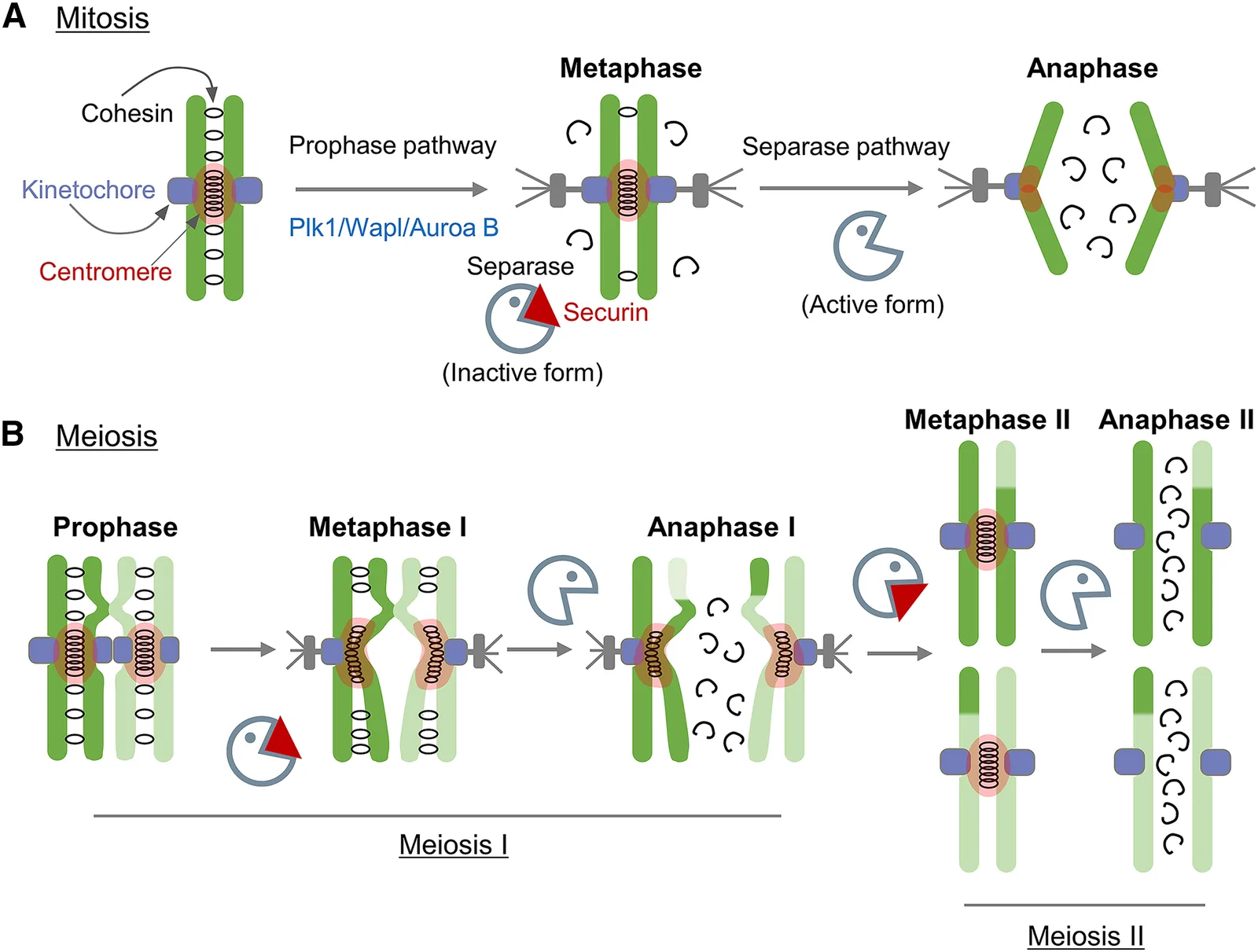
Chromosome segregation during mitosis and meiosis (A) Pathways to cohesin loss during mitosis. In prophase, cohesin dissociates from chromosome arms via WAPL. Upon chromosome biorientation, activated separase cleaves remaining cohesin, triggering chromosome segregation. (B) Spatial loss of cohesin during meiosis. Separase-dependent cohesin cleavage on chromosome arms during meiosis I resolves chiasmata and segregates homologous chromosomes. Protected pericentromeric cohesin maintains sister chromatid cohesion until meiosis II, where separase cleavage enables sister chromatid segregation. The prophase pathway protects centromeric cohesin through the SGO1-PP2A complex. At the metaphase-to-anaphase transition, separase cleaves the kleisin subunit, leading to cohesin release and sister chromatid segregation.

**Figure 4:**
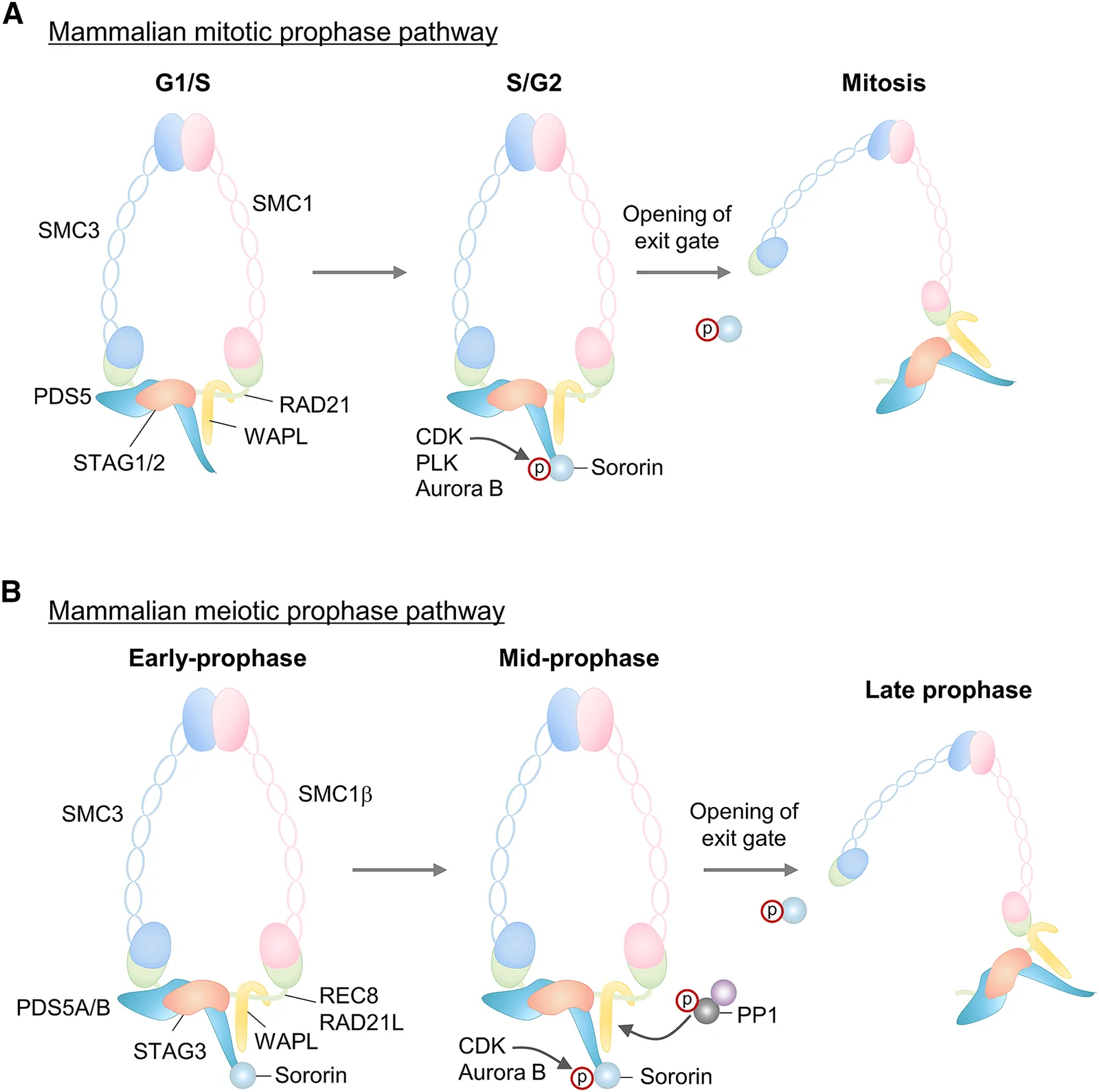
Cohesin composition and the prophase pathway for cleavage-independent cohesin removal (A) In mammalian cells, the cohesin ring entraps DNA (left). During late G2 and prometaphase, Sororin phosphorylation by PLK, Aurora B, and CDK induces its dissociation from cohesin (middle). Sororin release enables WAPL to open the SMC3-SCC1 exit gate, releasing entrapped DNA (right). (B) In mouse spermatocytes, RAD21L-containing cohesin is loaded onto DNA in a double-strand break-dependent manner (left). Nek1 kinase phosphorylates PP1γ, which dephosphorylates WAPL (middle). Dephosphorylated WAPL opens the exit gate (right).

These differences predict distinct mitotic-removal dynamics for any meiotic cohesin present in cycling ESCs. In particular, if REC8-STAG3 complexes in ESCs retain even partial sensitivity to SGO2-PP2A-mediated centromeric protection, this could in principle generate an aberrant, meiosis-like persistence of centromeric cohesin into mitotic anaphase.^12^^,^^42^ This abnormal retention would be expected to compromise chromosome segregation fidelity, showing as elevated rates of micronucleation, lagging chromosomes, or aneuploidy, phenotypes that warrant direct cytogenetic assessment in REC8/STAG3-expressing ESC populations.^58^^,^^70^ Conversely, if hybrid meiotic-mitotic complexes in ESCs are processed entirely through the canonical prophase-and-separase pathway irrespective of their REC8/STAG3, this would argue that centromeric protection is itself a meiosis-specific property requiring additional factors rather than an intrinsic property of the REC8 kleisin alone.^69^ Distinguishing between these hypotheses is essential both for interpreting the biological significance of meiotic-cohesin expression in pluripotent and reprogrammed cells, and for anticipating risks should meiotic cohesin genes be reactivated in therapeutic stem-cell products.^58^^,^^71^

## Expression and potential roles of meiotic cohesin factors in stem cells

Recent studies have reported detectable expression of meiosis-specific cohesin subunits, notably REC8 and STAG3, in pluripotent stem cells.^12^^,^^13^ Multiple groups have documented REC8/STAG3 transcripts and, in several cases, corresponding protein signals in ESCs, with biochemical evidence indicating REC8 and STAG3 co-association with core cohesin components such as SMC3 in ESCs but not in differentiated MEFs.^12^ These observations challenge the long-held assumption that meiotic cohesins are strictly restricted to germ cells and suggest that meiotic variants can be incorporated into cohesin complexes in mitotically dividing pluripotent populations.^15^^,^^58^

Expression analyses have been supported by proteomic and co-IP showing that REC8 and STAG3 form stable interactions with core cohesin subunits in the pluripotent state.^12^ In ESCs, REC8-STAG3 have been recovered in SMC3-containing complex, and ectopic expression of STAG3 can stabilize nuclear REC8 levels, consistent with a functional partnership between the two meiosis-specific subunits when present outside meiotic cells.^12^^,^^15^ The biochemical stability of REC8 in the presence of STAG3 indicates that meiotic cohesin components can assemble into competent cohesin rings in pluripotent cells rather than existing as isolated or non-functional polypeptides.^15^ Functional modulation studies have begun to uncover roles for meiotic cohesin factors in stem cell biology.^24^ RNAi or CRISPR-based depletion of REC8 or STAG3 in ESCs impairs replication fork progression, increases markers of replication stress and DNA damage, and alters the proportions of cells in S and M phases.^12^^,^^20^ Phenotypes are consistent with a role for REC8-STAG3 complexes in supporting replication dynamics and mitotic chromosome structure in rapidly cycling pluripotent cells.^13^^,^^19^ In addition, loss of STAG3 has been reported to influence the exit from pluripotency in mouse ESCs (mESCs), implicating meiotic cohesin subunits in processes that integrate cell-cycle and differentiation programs.^12^^,^^24^ Genome-wide chromatin profiling and imaging studies suggest that REC8/STAG3-containing cohesin complexes may occupy chromosomal locations that differ from, or partially overlap with, those bound by mitotic RAD21-cohesin.^16^^,^^72^ In ESCs, peaks of REC8/STAG3 enrichment have been observed at loci associated with active chromatin and at sites implicated in replication origin usage and sister-chromatid cohesion, implying that meiotic cohesins could influence both three-dimensional genome topology and replisome stability.^12^^,^^19^ Microscopy analyses further indicate that ESCs exhibit unique axis spacing and chromatin loop parameters relative to somatic cells, consistent with the presence of alternative cohesin species that modulate chromatin architecture in pluripotent nuclei.^13^^,^^73^

Several non-mutually exclusive models can account for how REC8 and STAG3 contribute to stem cell physiology. First, REC8 and STAG3 may provide topological constraints that favor chromatin configurations compatible with pluripotency-associated transcriptional programs, for example by altering loop extrusion kinetics or loop stability relative to RAD21-containing cohesion.^18^^,^^59^ Second, these meiotic variants may enhance replication-fork protection or facilitate replication-transcription interferences resolution during the rapid S-phases of ESCs, thereby reducing the frequency of fork collapse and DNA damage.^19^^,^^20^ Third, incorporation of meiotic subunits could confer different residence times or regulatory sensitivities-mediated via factors such as WAPL or Sororin—thereby expanding cohesin dynamics in cell types that require elevated chromatin flexibility.^15^^,^^21^ Evidence from studies in somatic systems supports the view that Rec8-containing cohesin requires STAG3 for function and is subject to cohesin regulators, lending to these models when manifested in pluripotent cells.^15^ Cellular and developmental assays further associate meiotic cohesin expression to stem cell function.^24^ Depletion of REC8 or STAG3 in ESCs leads to defects in proliferative capacity, an increase in mitotic abnormalities, and shifts in the balance between self-renewal and differentiation markers.^12^^,^^13^ In organoid or *in vivo* models where ESC-like progenitors contribute to tissue formation, REC8/STAG3 depletion alters the size of the progenitor pool and impacts tissue architecture.^24^^,^^74^ These findings are consistent with an intrinsic requirement for these subunits in maintaining genomic stability and precise lineage decisions.^20^

Comparative analyses across species and developmental stages indicate that REC8/STAG3 expression in mitotic cells is state dependent and regulated.^12^^,^^14^ While ESCs and early embryonic progenitors appear permissive for meiotic cohesin expression, most differentiated somatic cell types repress REC8/STAG3.^13^ The determinants of such susceptibility likely include transcriptional control of meiotic genes, post-translational stability conferred by STAG3, and differences in cohesin regulator abundance.^15^^,^^64^ The transient or low-abundance nature of meiotic cohesin in stem cells shows experimental challenges for detection and functional dissection, and supported the need to integrate biochemical, genomic and single-cell approaches to define the contributions of these subunits to stem cell chromatin biology.^75^^,^^76^

## Gene regulation by mitotic and meiotic cohesin

Beyond cohesion, the most important activity of cohesin in pluripotent cells is the spatial control of gene expression.^22^^,^^77^ In the subsequent section, we dissect how mitotic and meiotic cohesin regulate enhancer-promoter communication, how these activities are coupled to the shortened pluripotent cell cycle, and how the gene-regulatory networks of ESCs and MEFs diverge.^21^^,^^78^

### Cohesin-mediated loop extrusion and enhancer-promoter communication

Cohesin organizes the genome by extruding chromatin loops that are anchored, in large part, at convergent CTCF sites, thereby establishing TADs and bringing distal enhancers into proximity with their target promoters.^23^^,^^35^^,^^79^ The molecular mechanism of this process has been significantly clarified by single-molecule and cryo-EM studies.^38^^,^^39^ These studies show that cohesin, in complex with its loader NIPBL-MAU2, translocate along DNA in an ATP hydrolysis-dependent manner, stalling and stabilizing loops upon encountering a CTCF molecule bound in the convergent orientation.^38^^,^^80^ This stalling is mediated by interactions between the CTCF N-terminal tail and the NIPBL subunit of the cohesin loader, providing a structural explanation for why only convergently oriented CTCF sites function as robust loop anchors genome-wide (Figures 5A and 5B).^79^^,^^80^ Importantly, loop extrusion is not a binary process, as the size, stability, and transcriptional impact of a given loop domain are continuously modulated by cohesin processivity and the density of boundary elements, creating a quantitative rather than qualitative relationship between loop architecture and gene output.^18^^,^^81^

**Figure 5:**
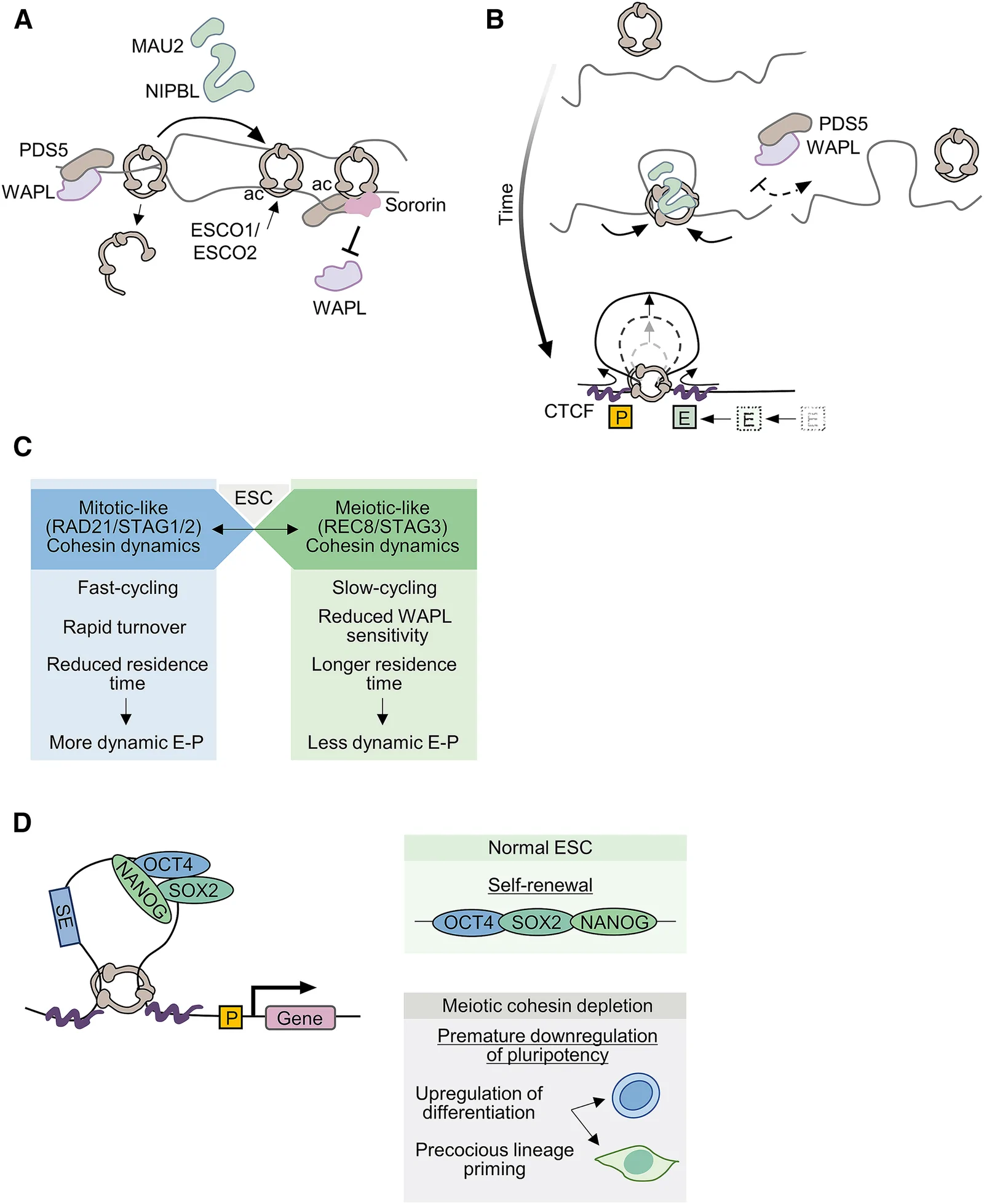
Cohesin-mediated loop extrusion (A) Sister chromatid cohesion mediated by cohesin. Dynamic cohesin bound to unreplicated chromatin dissociates via PDS5-bound WAPL. Assisted by NIPBL, cohesin embraces sister chromatids generated at the replication fork during S-phase. To prevent WAPL-mediated release, cohesin complexes are acetylated on SMC3 by ESCO1/2 and bound by Sororin via PDS5. (B) Role of cohesin in genome folding. In loop extrusion, cohesin binds chromatin and extrudes DNA with NIPBL until released by PDS5-WAPL, stalled by CTCF, or blocked by obstacles. Stable loops form between convergent CTCF sites. (C) Distinct dynamics of mitotic-like and meiotic-like cohesin complexes in ESCs. Mitotic-like cohesin exhibits fast-cycling dynamics, rapid turnover, and reduced chromatin residence time, promoting dynamic enhancer-promoter (E-P) interactions. In contrast, meiotic-like cohesin displays slow-cycling dynamics, reduced WAPL sensitivity, and longer residence time, resulting in less dynamic E-P interactions. (D) Functional impact of meiotic cohesin in pluripotency maintenance. Cohesin mediates SE (super-enhancer)-promoter looping in coordination with pluripotency transcription factors to drive gene expression. In normal ESCs, this arrangement supports self-renewal. Depletion of meiotic cohesin leads to premature downregulation of pluripotency, upregulation of differentiation genes, and precocious lineage priming.

The strength of these 3D interactions, set by cohesin processivity and genomic distance, directly tunes the quantitative effect of an enhancer on its target gene, such that loss of cohesin-mediated looping attenuates enhancer output.^82^^,^^83^ This quantitative relationship has been most directly demonstrated by combining auxin-inducible degron (AID) depletion of cohesin with paired enhancer-activity reporter assays, which reveal a near-linear relationship between ChIA-PET (chromatin interaction analysis by paired-end tag sequencing) contact frequency at a given enhancer-promoter pair and reporter expression level in the same cells.^83^^,^^84^ Loop domains are exquisitely cohesin-dependent.^37^ Indeed, acute cohesin depletion eliminates essentially all loop domains genome-wide within hours, demonstrating that cohesin is the principal motor of loop-based gene regulation and that passive thermal diffusion of distal elements is insufficient to sustain productive enhancer-promoter interactions at the distances typical of mammalian gene-regulatory topologies.^37^^,^^85^ Notably, compartment identity (A/B) is largely preserved upon acute cohesin loss, indicating that loop extrusion and compartment segregation are separable, and that the gene-regulatory function of cohesin is primarily mediated by intra-compartmental loop formation rather than by compartment switching.^37^^,^^86^

Cohesin is further stabilized at active promoters and enhancers, where it accumulates at *cis*-regulatory elements through interactions with Mediator, the general transcription machinery, and enhancer-bound transcription factors.^22^^,^^87^ Recent evidence suggests that RNA polymerase II elongation itself contributes to cohesin translocation in gene bodies, coupling transcriptional activity to loop dynamics in a positive feedback loop.^83^^,^^86^ Specifically, active transcription stabilizes cohesin at gene ends, which in turn promotes the enhancer-gene body associations that maintain transcriptional elongation.^83^ This interplay between cohesin dynamics and transcription conditions how meiotic cohesin variants function when incorporated into pluripotent chromatin.^59^ Thus, it remains unclear whether their altered binding durations and regulator interactions modify, promote, or redirect the loop-based logic established by canonical mitotic cohesin.^15^^,^^18^

### Meiotic cohesin and the maintenance of pluripotency transcriptional programs

Because REC8/STAG3 complexes occupy a partially distinct set of genomic sites, including developmental-regulatory loci, relative to RAD21 cohesin, they are positioned to modulate the transcriptional networks that sustain self-renewal.^12^^,^^16^ chromatin immunoprecipitation sequencing (ChIP-seq) and HiChIP analyses in ESCs reveal that REC8 and STAG3 co-occupy a subset of active enhancers and super-enhancers associated with key pluripotency regulators, including those controlling Oct4, Sox2, Nanog, and their downstream targets, at frequencies considerably exceeding what would be expected from random sampling of cohesin occupancy.^72^^,^^88^ The enrichment at super-enhancers is of particular interest given that these structures are thought to generate very robust transcriptional output through cooperative, condensate-like mechanisms.^88^^,^^89^ These elements are characterized by unusually dense clustering of enhancer elements, transcription-factor binding, and co-activator recruitment, with cohesin being required for their structural integrity.^82^ Consequently, any modification in cohesin occupancy time at these sites would be expected to have profound consequences for the transcription-factor networks they drive.^18^

The observation that STAG3 loss alters the exit from pluripotency in mESCs indicates that meiotic cohesin participates in the gene-regulatory decisions governing the self-renewal-versus-differentiation balance, not merely in structural maintenance.^12^^,^^24^ Specifically, cells depleted of STAG3 exhibit premature transcriptional downregulation of naive pluripotency markers and precocious upregulation of lineage-priming factors, consistent with a failure to maintain the regulatory contacts that sustain the naive transcriptional state.^12^ This phenotype is distinct from that produced by depletion of STAG2, which principally affects architectural TAD organization, suggesting that STAG3 is not simply redundant with STAG2 but instead mediates a distinct subset of regulatory loops that are disproportionately important for pluripotency gene expression.^16^^,^^90^ We propose that REC8/STAG3 complexes, owing to their longer chromatin residence times and extended loop architecture relative to canonical RAD21 cohesin, generate a subset of more stable enhancer-promoter interaction at pluripotency loci, thereby stabilizing transcription-factor networks against the rapid loop turnover imposed by the ESC cell cycle.^15^^,^^18^^,^^88^

This model is supported by reports that long-range loop formation supports stem-cell pluripotency and that distinct STAG identities segregate cohesin between architectural and regulatory functions.^31^^,^^88^ This model is further supported by super-enhancer condensates (Figures 5C and 5D).^89^ Just as liquid-liquid phase separation at super-enhancers has been proposed to render pluripotency gene expression to fluctuations in transcription-factor concentration, long-lived cohesin-mediated chromatin loops might render the same loci immune to cell-cycle-associated cohesin stripping.^18^^,^^89^ This process ultimately provides a structural memory mechanism that transmits regulatory interactions across generation.^91^ Validating this hypothesis will require high-resolution, allele-specific Hi-C or Micro-C in isogenic ESC lines carrying acute-depletion alleles of REC8 and STAG3, ideally combined with single-molecule live-cell imaging to directly measure cohesin occupancy times at pluripotency super-enhancers in cycling cells.^68^^,^^92^^,^^93^

### Cell-cycle coupling of cohesin-dependent gene regulation in pluripotent cells

Pluripotent stem cells divide with unusually rapid cycles, approximately 10–12 h in mESCs and 16–18 h in hESCs compared with approximately 24 h in typical somatic cells, and are distinguished by markedly shortened G1 phases, spending the majority of their cycle in S and G2/M.^24^ This shortened G1 is thought to restrict the window in which lineage-specifying transcription-factor networks can be stably assembled, and has been proposed to contribute to the pluripotent state by limiting the opportunity for cells to commit to differentiation.^94^ However, this time constraint creates a parallel problem for cohesin-dependent genome organization, since cohesin is dynamically loaded during G1, stripped from chromatin by the prophase pathway and WAPL-mediated release throughout the cell cycle, and then re-establishes cohesion in the subsequent S-phase.^21^^,^^41^^,^^91^ As a result, the compressed G1 phase in ESCs substantially shortens the window in which cohesin can organize regulatory loops before the next round of loop dissolution begins.^91^ This temporal compression imposes a dual constraint, by requiring that cohesin must simultaneously support high-fidelity DNA replication and continuously re-establish the enhancer-promoter contacts that maintain pluripotency gene expression.^19^ Mitotic RAD21 is dynamically removed and reloaded each cycle, so loop-encoded regulatory information must be re-established after every division.^4^^,^^68^ Several model of cohesin dynamics in rapidly cycling cells predicts a reduction in mean loop occupancy time and average loop size relative to slowly cycling cells, particularly for loops anchored by weak or intermediate-affinity CTCF sites, precisely the category of loop that would severely affect distal enhancer-promoter contacts across the relatively large TADs associated with developmental loci.^18^^,^^81^ Given this background, the incorporation of meiotic REC8/STAG3 complexes provides a complementary, longer-lived pool of cohesin that helps preserve key regulatory contacts across the rapid cell cycle, partially decoupling gene-regulatory architecture from the cohesin-stripping events of each mitosis.^12^^,^^15^

This decoupling function could operate through at least two non-mutually exclusive mechanisms. First, the altered WAPL-binding interface of REC8- and STAG3-containing complexes may reduce the rate of cohesin release during the prophase pathway, creating a population of chromatin-resident cohesin that persists into late G2 and early mitosis and re-seeds loop formation more rapidly after anaphase.^15^^,^^36^ Second, meiotic cohesin complexes in ESCs may exhibit reduced sensitivity to the prophase pathway relative to RAD21 cohesin, as predicted from their structural separation and meiosis-specific protection mechanisms.^42^^,^^69^ Consequently, these complexes could serve as persistent loop anchors that survive mitosis, thereby providing spatial memory for loop re-establishment in the subsequent G1 phase, even within a shortened cell cycle.^18^^,^^91^ The replication-stress and S/M-phase distribution phenotypes observed upon REC8/STAG3 depletion are consistent with such a cell-cycle-coupled, dual structural/regulatory role.^12^^,^^20^ Cohesin depletion under these conditions may simultaneously impair replication-fork stability, a well-established role for cohesin in protecting stalled forks, and de-stabilize the transcriptional circuits that promote balanced replication-origin activation across the pluripotent genome.^13^^,^^19^

### Differential gene regulation in ESCs versus MEFs

Evidence for cell type-dependent meiotic-cohesin function comes from the ESC versus MEF comparison.^12^^,^^13^ REC8 and STAG3 are expressed and chromatin-associated in ESCs but are largely repressed in differentiated MEFs, and they co-precipitate with SMC3 only in the pluripotent state.^12^ Analysis confirms that meiotic-cohesin expression is a regulated feature rather than a leaky developmental byproduct. Instead, it represents a consistent characteristic of the ESC state.^13^ Specifically, REC8 and STAG3 protein levels in ESCs are detectable by standard immunoblot and quantitative mass spectrometry, with STAG3 abundance approaching that of STAG1 and STAG2 in some ESC preparations, whereas both proteins fall below detection limits within days of LIF withdrawal or following ectopic expression of lineage-specifying transcription factors.^12^^,^^66^

This differential expression translates onto differential genome organization.^16^ Specifically, ESCs display distinct chromatin-axis spacing and loop parameters relative to somatic cells, and the cohesin-STAG1/STAG2 balance itself reorganizes TADs and Polycomb domains specifically in the pluripotent state.^16^^,^^95^ Thus, the gene regulatory deployment of cohesin is cell-state-specific at two structural levels, which include the meiotic and mitotic subunit choice and the STAG1, STAG2, or STAG3 identity.^5^^,^^31^ Each of these choices is reset during the transcriptional transition from pluripotency to somatic commitment.^94^ Importantly, the effects of shifting the STAG balance are not homogenous across the genome.^16^ Loci associated with bivalent chromatin marks (H3K4me3/H3K27me3), which are characteristic of developmentally poised genes in ESCs but resolved to monovalent states upon differentiation, show pronounced sensitivity to STAG identity, likely because the competing cohesin species differentially modulate the interactions between Polycomb-repressive domains and their regulated targets.^90^^,^^95^ The suppression of REC8/STAG3 as ESCs become MEFs therefore represents a regulated switch in cohesin composition that accompanies, and may help impose, the transcriptional transition out of pluripotency.^12^^,^^13^ This switch is interesting because it implies that the differentiation program must silence a cohort of genes (REC8, STAG3, SMC1B) that are actively transcribed in the pluripotent state and whose continued expression in differentiated cells would, according to the model developed earlier, maintain a more stable regulatory loop configuration incompatible with the lineage-restricted transcriptional landscape characteristic of somatic identity.^77^^,^^94^ Accordingly, ectopic expression of REC8 and STAG3 in MEFs partially resists transcriptional silencing at a subset of pluripotency-associated loci and attenuates the efficiency of direct reprogramming, suggesting that cohesin-composition switching is not merely a consequence of differentiation but a mechanistic contributor to the establishment and maintenance of somatic transcriptional identity.^58^^,^^71^ Whether targeted modulation of cohesin subunit composition, for instance, by transiently suppressing REC8/STAG3 in partially reprogrammed intermediates, could accelerate or improve induced pluripotent stem cell (iPSC) generation is a therapeutic hypothesis of considerable practical interest that remains to be validated in reprogramming experiments.^71^^,^^96^

## Cohesin interacts with transcription factors to control cell type-specific enhancers

Whether cohesin shapes the regulatory architecture by establishing chromatin loops that bring enhancers and promoters into proximity, or whether active *cis*-regulatory elements direct cohesin to specific genomic positions and thereby instruct loop formation, remains unclear.^22^^,^^87^ This shows that cohesin acts downstream of transcription factors, explaining how meiotic cohesin variants are distributed in pluripotent cells.^97^

### Active regulatory elements recruit cohesin to build cell-specific chromatin domains

Whether cohesin establishes chromatin loops to bring enhancers and promoters into proximity, or active *cis*-regulatory elements direct cohesin to specific genomic positions to instruct loop formation, remains unclear.^98^ Active *cis*-regulatory elements act upstream of cohesin, recruiting it to defined genomic positions to nucleate cell type-specific loop domains.^87^^,^^97^

The alpha-globin locus has emerged as a suitable model for dissecting this mechanism.^98^ Using allele-specific ChIP-seq in primary human erythroblasts, cohesin occupancy correlated closely with enhancer activity and varied quantitatively at positions carrying polymorphic enhancer variants, with the stronger enhancer allele recruiting accordingly more cohesin in *cis*.^87^ This allele specificity was observed even where both alleles shared identical CTCF binding profiles, demonstrating that interior cohesin recruitment is driven by enhancer activity rather than by flanking boundary architecture.^87^^,^^99^ When all enhancers within the cluster were deleted, cohesin peaks vanished from the locus interior and the erythroid-specific sub-TAD failed to form, while CTCF-bound TAD boundaries remained stable.^100^ This dissociation indicates that CTCF boundaries define a permissive topological domain within which enhancer-driven cohesin recruitment organizes cell type-specific sub-loops.^79^^,^^98^ Conversely, inserting the R2 enhancer into a neutral genomic region was sufficient to recruit cohesin *de novo*, activate nearby transcription, and generate a new ∼75 kb erythroid-specific chromatin domain that replicated the key features of the endogenous alpha-globin sub-TAD.^100^ The specificity of this effect to erythroid cells confirmed that a cohesin-nucleating enhancer requires the appropriate cell-type-specific transcription factor to function, rather than acting through any structural property of the DNA sequence itself.^101^

The molecular mechanism coupling enhancer activity to cohesin recruitment likely involves Mediator, which bridges transcription factors to RNA polymerase II and physically interacts with cohesin, as well as BRD4, which interacts with NIPBL-MAU2 loader components at active enhancers.^22^^,^^89^ Computational analyses integrating cell-type-specific chromatin-state features can predict cohesin-mediated loops with high accuracy without prior knowledge of interaction maps.^93^^,^^102^ This demonstrates that the *cis*-regulatory logic governing cohesin positioning is broadly shared across lineages.^78^ The 3D consequences of non-coding GWAS (genome-wide association studies) variants that alter enhancer activity can be traced through their downstream effect on cohesin recruitment.^103^

### Lineage-determining transcription factors constrain cohesin to drive cell-type-specific folding

Lineage-determining transcription factors actively restrict where cohesin stalls and accumulates, shaping how individual chromatin fibers fold to connect distant enhancers with key genes.^97^^,^^101^ Master transcription factors establish CTCF-independent boundaries and anchor points for loop extrusion, generating the lineage-specific genome folding that distinguishes cell types.^78^^,^^97^ Cohesin is also stabilized at promoters and enhancers by common regulatory inputs, providing a mechanism by which transcription factor-bound elements convert into durable loop anchors.^87^ Core pluripotency transcription factors define the cohesin-bound enhancer-promoter network to maintain the stem-cell state, while REC8/STAG3 complexes recruited to these elements impart distinct stabilities to pluripotency-specific interactions.^12^^,^^88^

### WAPL-controlled cohesin cycling preserves distal gene regulation

The gene-regulatory output of cohesin depends not only on where it is loaded but on how rapidly it is released.^21^ WAPL maintains a cohesin loading/unloading cycle that is required to preserve cell-specific distal gene regulation, such that inhibiting WAPL, and thereby extending cohesin residence, disrupts the normal enhancer-promoter interaction of a given cell type.^21^^,^^36^ This is directly relevant to meiotic cohesin: because REC8/STAG3 complexes display altered WAPL/Sororin release kinetics and longer binding time than RAD21 cohesin, their incorporation in ESCs effectively introduces a second, slower cohesin cycle.^15^^,^^18^ We propose that the coexistence of fast-cycling RAD21 and slower-cycling REC8/STAG3 cohesin allows pluripotent cells to maintain stable core enhancer-promoter interactions while preserving the flexibility to reorganize regulatory loops during differentiation (Figures 5C and 5D).^12^^,^^78^ This model can be verified by allele-specific, cell-cycle-resolved ChIP-seq of RAD21 and REC8 at enhancers.^104^

### Integrating transcription factor-guided cohesin recruitment with meiotic cohesin in pluripotency

Cohesin’s regulatory output at any given locus is jointly determined by where the complex is loaded, where its translocation is arrested, and how long it persists once positioned.^18^^,^^80^ Active *cis*-regulatory elements and lineage-determining transcription factors together define the genomic coordinates of cohesin occupancy, while the loading-unloading cycle governed by WAPL sets the residence time available for enhancer-promoter contacts to mature into stable, functional loops.^21^^,^^87^ However, this model has ignored the biochemical identity of the cohesin complex.^59^

Somatic and meiotic cohesin share a tripartite core but differ in release kinetics.^15^ REC8/STAG3 complexes exhibit significantly delayed WAPL/Sororin-mediated release and longer chromatin binding times than RAD21 cohesin.^15^^,^^18^ This implies that pluripotent cells operate two distinct cohesin populations in parallel, a fast-cycling RAD21 pool and a slow-cycling REC8/STAG3 pool, rather than a single homogeneous pool.^12^ We propose that this dual-cycle architecture is partitioned by regulatory demand. Enhancer-promoter interactions within the core pluripotency networks, those bound by OCT4, SOX2, and NANOG at loci required for self-renewal, depend on persistent and repeated interactions.^88^ Selective stabilization of REC8/STAG3 at these positions would provide a mechanism for maintaining such loops over extended periods, effectively fixing the core regulatory architecture in place.^18^ Conversely, the fast-cycling RAD21 pool retains the flexibility needed to rapidly reorganize loops as lineage specific transcription factors appear during differentiation.^21^^,^^97^ Under this model, REC8/STAG3 stabilizes essential structures, while RAD21 turnover allows structural changes.^15^

This hypothesis generates several testable predictions. First, REC8/STAG3 occupancy, measured by ChIP-seq, should be enriched at pluripotency-specific super enhancers and their target promoters.^72^^,^^104^ Second, allele-specific, cell-cycle-resolved ChIP-seq performed in parallel for RAD21 and REC8 should reveal selectively extended residence times at REC8-bound sites relative to RAD21 signal at the same loci.^68^^,^^104^ Third, acute depletion of REC8 or STAG3, measured by Hi-C or HiChIP, should selectively reduce interaction frequency at core pluripotency enhancer-promoter loops, while leaving CTCF-anchored boundaries and lineage-nonspecific loops unaffected.^37^^,^^85^ Fourth, if the relationship between enhancer activity and cohesin occupancy generalizes to pluripotent cells, then CRISPR interference or activation at enhancers should proportionally modulate REC8/STAG3 occupancy at their corresponding loop anchors.^103^

This model has implications beyond chromatin biology.^48^ Inherited variants in cohesin loader and core subunit genes because developmental disorders termed cohesinopathies, typically interpreted through CTCF-anchored structural defects and global loading deficiency.^33^^,^^48^^,^^105^ If REC8/STAG3 performs a distinct regulatory function in pluripotent cells, this complex may constitute an additional axis governing pluripotency stability during early embryogenesis and stem cell-derived therapeutic manufacturing.^13^^,^^24^ Here, modulating REC8 or STAG3 expression could improve reprogramming efficiency or pluripotency robustness in cell therapy production processes.^71^^,^^96^ In addition to its role in protecting replication forks during rapid proliferation, meiotic cohesin emerges as a dual-function module adapted to maintain both genome stability and regulatory networks in the pluripotent state.^19^^,^^20^

Collectively, these observations indicate that cohesin subunit identity, RAD21 versus REC8/STAG3, determines regulatory activity by controlling chromatin binding affinity.^15^^,^^18^ REC8/STAG3 expression in pluripotent cells expands the cohesin recruitment machinery, selectively stabilizing specific pluripotency-associated enhancer-promoter interactions while protecting replication forks in dividing cells.^12^^,^^19^ Meiotic cohesin is therefore an active contributor to cell-specific genome regulation.^24^

## Discussion

The evidence summarized in this review supports an emerging paradigm in which meiotic cohesin subunits, particularly REC8 and STAG3, are not strictly confined to the meiotic processes but instead contribute to chromosome biology in pluripotent stem cells.^12^^,^^13^ This finding challenges the opposition between mitotic and meiotic cohesin systems and raises important questions regarding the functional plasticity of SMC complexes.^1^^,^^3^

A central theme arising from the available data is that REC8 and STAG3 can link into competent cohesin complexes in ESCs, as demonstrated by co-IP with core subunits such as SMC3.^12^ The observation that STAG3 stabilizes nuclear REC8 levels suggests that these meiosis-specific proteins retain their characteristic partnership even when expressed outside their meiotic process.^15^ This biochemical compatibility implies that the cohesin ring architecture is flexible to accommodate kleisin and STAG subunits, potentially generating cohesin variants with distinct residence times, regulatory sensitivities, or substrate specificities.^18^^,^^59^ Whether such alternative complexes coexist with RAD21-STAG1/2 cohesin within the same cell or are expressed in distinct subpopulations of pluripotent cells remains an open question that warrants further investigation using single-cell approaches.^14^

This comparative analysis clarifies that meiotic and mitotic cohesin are best understood as variations on a conserved loop-extruding machine that are optimized for different biological function.^2^^,^^3^ Both complexes share the same core mechanism of NIPBL, WAPL, PDS5, and Sororin-regulated ATP-driven loop extrusion.^23^^,^^38^ However, replacing RAD21 with REC8, STAG1/2 with STAG3, and SMC1A with SMC1B alters cohesin chromatin retention, dissociation pathways, and loop size.^15^^,^^56^ Remarkably, a complex evolved for meiotic chromosome segregation can be reorganized in pluripotent cells to protect replication forks and stabilize specific gene regulatory interactions.^12^^,^^19^ This functional adjustment serves as the primary focus of this review, moving beyond the simple presence of meiotic subunits in stem cells.^24^

The recruitment of cohesin by active enhancers and lineage-determining transcription factors to shape cell-specific domains redefines the role of meiotic cohesin in pluripotency.^87^^,^^97^ Cohesin occupancy tracks active *cis*-elements in an allele-specific manner, and removing or inserting enhancers abolishes or creates cohesin-anchored, cell-specific sub-TADs, establishing that regulatory elements causally direct cohesin positioning.^87^^,^^100^ Lineage-determining transcription factors then constrain cohesin to fold individual fibers into cell-specific configurations, and the WAPL-controlled loading cycle preserves the resulting distal gene regulation.^21^^,^^101^ Because REC8/STAG3 complexes have lower WAPL/Sororin sensitivity and longer binding times, they create a slower-cycling cohesin pool.^15^^,^^18^ This pool stabilizes enhancer-promoter interactions essential for pluripotency, explaining why STAG3 loss disrupts the exit from pluripotency.^12^^,^^88^

The functional significances of meiotic cohesin expression in stem cells appear to be 2-fold.^13^ First, REC8/STAG3-containing complexes may modulate higher-order chromatin architecture by altering loop extrusion dynamics or cohesin turnover rates.^17^^,^^18^ Given that pluripotent cells maintain a uniquely open and dynamic chromatin state to support broad transcriptional plasticity, the incorporation of meiotic cohesin variants could provide a layer of architectural regulation that enhancer-promoter interactions.^66^^,^^77^ The partial overlap of REC8/STAG3 binding sites with cohesin targets, combined with their enrichment at developmental regulatory loci, is consistent with a model in which these factors contribute to the maintenance of pluripotency-associated transcriptional programs.^12^^,^^16^ Second, the association of meiotic cohesin depletion with elevated replication stress, DNA damage markers, and mitotic abnormalities implicates these subunits in genome stability pathways.^12^^,^^20^ Pluripotent stem cells are characterized by unusually rapid cell cycles with shortened G1 and G2 phases, which impose significant demands on replication machinery and DNA repair systems.^24^^,^^94^ Thus, REC8/STAG3 complexes may facilitate replication fork protection, promote efficient sister chromatid cohesion establishment during S-phase, or contribute to the resolution of replication-transcription interference.^13^^,^^19^ The recruitment of meiotic cohesin to stalled replication forks, as reported in some studies, provides a potential mechanistic basis for these protective functions.^19^^,^^20^ It is tempting to risk that the retention of meiotic cohesin expression in pluripotent cells reflects an adaptation to the heightened genomic stress associated with rapid proliferation.^20^

Several mechanistic questions follow directly from this model. First, do RAD21 and REC8 cohesin occupy the same transcription-factor-bound enhancers, or do they partition between regulatory and protective genomic compartments? Allele-specific, cell cycle-resolved CUT&Tag (cleavage under targets and tagmentation) for RAD21 versus REC8, integrated with Micro-C, would resolve this.^75^^,^^76^^,^^106^^,^^107^ Second, are the gene-regulatory and genome-stability functions of REC8/STAG3 separable, for example, through separation of function alleles that disrupt loop stabilization without affecting fork protection?^18^^,^^59^ Third, how is the meiotic-cohesin program silenced during the ESC to MEF transition, and does forced REC8/STAG3 expression in somatic cells reorganize their enhancer-promoter networks?^12^^,^^58^ Finally, given that ectopic meiotic cohesin can generate chromosome instability in cancer cells, does the same machinery that protects pluripotent genomes become pathogenic when mis-expressed during tumorigenesis or reprogramming?^47^^,^^58^

Several technical considerations merit attention when interpreting the findings.^14^ The detection of meiotic cohesin expression in pluripotent cells often relies on transcriptomic or proteomic analyses that may be confounded by population heterogeneity, low expression levels, or antibody cross-reactivity.^14^^,^^65^ The development of more sensitive and specific detection methods, including epitope-tagged knock-in alleles and single-molecule imaging approaches, will be essential for resolving questions about the abundance, localization, and dynamics of these proteins.^68^^,^^73^ Furthermore, the use of asynchronous cell populations in many chromatin profiling studies complicates the interpretation of cohesin and condensin co-occupancy, as apparent overlap may reflect sequential rather than simultaneous binding events.^92^^,^^108^

In conclusion, the unexpected presence and functional relevance of meiotic cohesin components in pluripotent stem cells expand our understanding of SMC complex biology and suggest that the boundary between meiotic and mitotic chromosome organization is more permeable than previously appreciated.^1^^,^^12^ By comparing the two complexes directly and by situating meiotic cohesin within the transcription-factor-guided logic of cell-specific enhancer regulation, we propose that REC8/STAG3 complexes act as non-canonical, slower-cycling regulators of pluripotent genome folding and stability.^15^^,^^18^ Continued investigation, particularly allele and cell-cycle-resolved mapping of meiotic versus mitotic cohesin at active enhancers, promises new insights into the mechanisms that govern chromatin architecture, gene expression, genome stability, and cell fate decisions in stem cells and beyond.^24^^,^^104^

## Acknowledgments

This research was supported by the Regional Innovation System & Education (RISE) program through the Chungbuk RISE Center, funded by the Ministry of Education (MOE) and the Chungcheongbuk-do, Republic of Korea (2025-RISE-11-004).

## Author contributions

S.M.C., Y.E.K. and E.-H.C. contributed equally to this manuscript; S.M.C., Y.E.K., and E.-H.C. completed literature review, investigation, wrote the original draft, and designed the figures; S.M.C. and E.-H.C. conceived the study, revised the draft, and provided funding acquisition and supervision. All the authors approved the submission.

## Declaration of interests

The authors declare no conflict of interest.
